# Dye Sensitized Solar Cells

**DOI:** 10.3390/ijms11031103

**Published:** 2010-03-16

**Authors:** Di Wei

**Affiliations:** NOKIA Research Centre c/o University of Cambridge, Broers Building, 19 JJ Thompson Avenue, CB3 0FA, Cambridge, UK; E-Mail: di.wei@nokia.com

**Keywords:** photoelectrochemistry, dye sensitized solar cells (DSSC)

## Abstract

Dye sensitized solar cell (DSSC) is the only solar cell that can offer both the flexibility and transparency. Its efficiency is comparable to amorphous silicon solar cells but with a much lower cost. This review not only covers the fundamentals of DSSC but also the related cutting-edge research and its development for industrial applications. Most recent research topics on DSSC, for example, applications of nanostructured TiO_2_, ZnO electrodes, ionic liquid electrolytes, carbon nanotubes, graphene and solid state DSSC have all been included and discussed.

## 1. Introduction

Photoelectrochemical solar cell is generally composed of a photoactive semiconductor working electrode and counter electrode made of either metal (e.g., Pt) or semiconductors. Both electrodes are immersed in the electrolyte containing suitable redox couples. If the semiconductor-electrolyte interface (SEI) is illuminated with a light having energy greater than the bandgap of the semiconductor, photogenerated electrons/holes are separated. The photogenerated minority carriers arrive at the interface of the semiconductor-electrolyte. Photogenerated majority carriers accumulate at the backside of the semiconductor. With the help of a charge-collecting substrate, photogenerated majority carriers are transported *via* a load to the counter electrode where these carriers electrochemically react with the redox electrolyte. A pioneering photoelectrochemical experiment was realized by obtaining photocurrent between two platinum electrodes immersed in the electrolyte containing metal halide salts [1]. It was later found that the photosensitivity can be extended to longer wavelengths by adding a dye to silver halide emulsions [2]. The interest in photoelectrochemistry of semiconductors led to the discovery of wet-type photoelectrochemical solar cells [3–5]. Grätzel has then extended the concept to the dye sensitized solar cells (DSSC) by adsorption of dye molecules on the nanocrystalline TiO_2_ electrodes.

## 2. Dye Sensitized Solar Cells (DSSCs)

DSSCs differ from conventional semiconductor devices in that they separate the function of light absorption from charge carrier transport. Dye sensitizer absorbs the incident sunlight and exploits the light energy to induce vectorial electron transfer reaction. Thus DSSCs have the following advantages comparing with the Si based photovoltaics. (1) It is not sensitive to the defects in semiconductors such as defects in Si. (2) The SEI is easy to form and it is cost effective for production. (3) It is possible to realize the direct energy transfer from photons to chemical energy. The earlier photoelectrochemical studies of dye sensitization of semiconductors focused on flat electrodes, but these systems were facing an intrinsic problem [6]. Only the first monolayer of adsorbed dye results in effective electron injection into the semiconductor, but such light-harvesting from a single dye monolayer is extremely small. By application of nanoporous TiO_2_, the effective surface area can be enhanced 1000-fold. An intriguing feature in the nanocrystalline TiO_2_ film is that the charge transport of the photo-generated electrons passing through all the particles and grain boundaries is highly efficient [7]. Solar cell based on a dye sensitized porous nanocrystalline TiO_2_ photoanode with attractive performance was first reported by Grätzel *et al.* [8,9]. Interest in nanoporous semiconductor matrices permeated by an electrolyte solution containing dye and redox couples has been stimulated by their reports. The power conversion efficiency of the DSSC has been currently improved to 11.5% [10] since the first DSSC was reported with efficiency of 7.1% [9], comparable with the amorphous Si cells [11]. Large-size DSSC has been prepared on silver grid embedded fluorine-doped tin oxide (FTO) glass substrate by screen printing method [12]. Under the standard test condition, energy conversion efficiency of active area was achieved to 5.52% in 5 cm × 5 cm device, which is comparable to 6.16% of small-size cell prepared at similar condition. G24 Innovation Ltd., based on the technology invented by Grätzel, uses a low-cost, roll-to-roll process to make its flexible DSSC modules, which produce 0.5 watts of power under direct sunlight. Miyasaka *et al.* developed a 2.1 m × 0.8 m DSSC module by connecting eight pieces of 10 cm^2^ panels with six embedded cells. The module conversion efficiency is expected to be approximately 3% and was displayed at the 1^st^ International Photovoltaic Power Generation Expo in 2008.

**Scheme 1.**
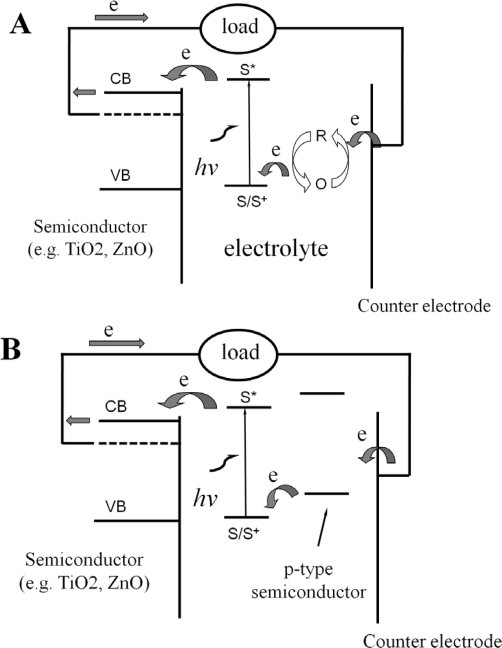
Operation mechanism of the dye sensitized electrochemical solar cell (DSSC). S: Dye sensitizer, S^*^: Electronically excited dye sensitizer, S^+^: oxidized dye sensitizer O/R: redox couple (e,g, I_3_^−^/I^−^). CB: Conduction band for semiconductors, VB: valence band for semiconductors. (A) Wet-type DSSC with redox couple in the liquid electrolyte. (B) Solid state DSSC with a p-type semiconductor to replace the electrolyte containing the redox couple.

In DSSC, the initial photoexcitation occurs in the light absorbing dye as shown in Scheme 1. Nanoporous semiconductors such as TiO_2_ not only act as support for dye sensitizer but also function as electron acceptor and electronic conductor. Subsequent injection of electrons from the photo-excited dye into the conduction band of semiconductors results in the flow of current travelling across the nanocrystalline TiO_2_ film to the charge collecting electrode and then to the external circuit. Sustained conversion of light energy is facilitated by regeneration of the reduced dye sensitizer either *via* a reversible redox couple (O/R), which is usually I_3_^−^/I^−^ (Scheme 1A) or *via* the electron donation from a p-type semiconductor (Scheme 1B).

Scheme 1A shows the mechanism of a traditional wet-type DSSC containing redox couples in electrolyte. The photoanode, made of a nanoporous dye-sensitized n-type semiconductor, receives electrons from the photo-excited dye sensitizer which is thereby oxidized to S^+^. The neutral dye sensitizer (S) can be regenerated by the oxidation reaction (R→O) of the redox species dissolved in the electrolyte. The mediator R will then be regenerated by reduction at the cathode (O→R) by the electrons circulated through the external circuit.

The need for DSSC to absorb far more of the incident light was the driving force for the development of mesoscopic semiconductor materials with an enormous internal surface area. The major breakthrough in DSSC was the use of a high surface area nanoporous TiO_2_ layer. A single monolayer of the dye on the semiconductor surface was sufficient to absorb essentially all the incident light in a reasonable thickness (several um) of the semiconductor film. TiO_2_ became the semiconductor of choice with advantage properties of cheap, abundant, and non-toxic [14]. The choice of dye is also an important parameter. The first organic-dye photosensitization was reported in 1887 [13]. In traditional DSSC, the standard dye was tris(2,2′-bipyridyl-4,4′-carboxylate)ruthium (II) (N_3_ dye). The function of the carboxylate group in the dye is to attach the semiconductor oxide substrate by chemisorption [14]. The dye must carry attachment groups such as carboxylate or phosphonate to firmly graft itself to the TiO_2_ surface. The attachment group of the dye ensures that it spontaneously assembles as a molecular layer upon exposing the oxide film to a dye solution. It will make a high probability that, once a photon is absorbed, the excited state of the dye molecule will relax by electron injection to the semiconductor conduction band. The photovoltaic performance of N_3_ dye has been irreplaceable by other dye complexes since 1993 [15]. A credible challenger was identified with tri(cyanato-2,2′,2″-terpyridyl-4,4′,4″-tricarboxylate) Ru (II) (black dye) [8], whose response extends 100 nm further into the IR than that of the N_3_ dye [16]. It is not until recently that a high molar extinction coefficient heteroleptic ruthium complex has been synthesized and demonstrated as more efficient sensitizer for DSSCs [10].

Because of the encapsulation problem posed by the use of liquid in the conventional wet-type DSSC, much work is being done to make an all solid state DSSC [17,18]. The use of solvent free electrolytes in the DSSC is supposed to offer very stable performance for the device. To construct a full solid-state DSSC, a solid p-type conductor should be chosen to replace the liquid electrolyte. The redox levels of the dye and p-type materials have to be adapted carefully as Scheme 1B shows. It results in an electron in the conduction band of n-type semiconductors (e.g., TiO_2_) and a hole localized on the p-type conductor. Hole transporting amorphous materials have been used in nanocrystalline TiO_2_ based DSSC to transport hole carriers from the dye cation radical to the counter electrode instead of using the I_3_^−^/I^−^ redox species [17,19]. Early work focused on the replacement of I_3_^−^/I^−^ liquid electrolyte with CuI. CuI as a p-type conductor, can be prepared by precipitation from an acetonitrile solution at room temperature and it is also a solid state ionic conductor. Cells made this way gave solar efficiencies of several percent, but their stability is relatively poor due to the liability of CuI to air and light [18]. Besides CuI, CuSCN has also been tried [20,21]. Organic hole transporting materials will offer flexibility and easier processing. Bach *et al.* used a hole conducting amorphous organic solid deposited by spin coating [17]. However, deposition in nanoporous materials cannot be easily achieved by traditional methods such as evaporation or spin coating. Electrochemical deposition of organic semiconductors on high surface area electrodes for solar cells has also been described [22]. A thin layer of organic semiconductors can be electrochemically deposited on a nanoporous TiO_2_ electrode.

**Figure 1.**
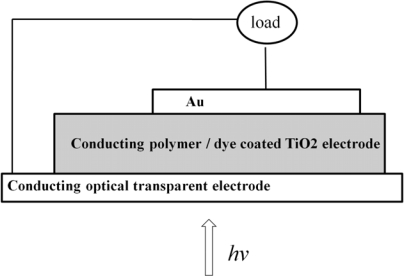
The prototype solid state DSSC.

One of the first solid state dye sensitized heterojunctions between TiO_2_ and conducting polymer was reported by Murakoshi and coworkers [23]. The prototype of this kind of solid state DSSC is shown in Figure 1.

Conducting polymer such as pyrrole was electrochemically polymerized on porous nanocrystalline TiO_2_ electrode, which was sensitized by N_3_ dye. Polypyrrole successfully worked as a hole transport layer connecting dye molecules anchored on TiO_2_ to the counter electrode. Conducting polyaniline has also been used in solid state solar cells sensitized with methylene blue [24]. This solid state DSSC was fabricated using conducting polyaniline coated electrodes sandwiched with a solid polymer electrolyte, poly(vinyl alcohol) with phosphoric acid. It exhibits good photoresponse to visible light. The presence of illumination enhances the electrochemical reaction (doping of polyaniline by migration of anions). The observed I-V characteristics are the superposition of the Ohmic charge transport and the electrochemical reaction. Recently, a low bandgap polymer consisting of alternating thiophene and benzothiadiazole derivatives was used in the bulk heterjunction DSSC. This solid state DSSC using conducting polymer exhibited a power conversion efficiency of 3.1% [25]. To date, the highest power conversion efficiency value with organic hole-transport materials in DSSC is over 5%, reported by Snaith *et al.* [26].

Construction of quasi-solid-state DSSC has also been explored. Quasi-solid-state DSSCs can be made based on the polymer grafted nanoparticle composite electrolyte [27], cyanoacrylate electrolyte matrix [28], and a novel efficient absorbent for liquid electrolyte consisting poly(acrylic acid)-poly(ethylene glycol) hybrid [29]. The polymer gels in above cases function as ionic conductors. Room temperature ionic liquids are also known as good ionic conductors [30,31]. DSSCs using imidazolium type ionic liquid crystal systems as effective electrolytes were reported [32]. Solid state DSSCs based on ionic liquids were reported to enhance the conversion efficiency of DSSCs [33]. Ionic liquid oligomers, which were prepared by incorporating imidazole ionic liquid with polyethylene oxide oligomers have also been tried as electrolyte for DSSC [34]. It shows that the increase of the polyethylene oxide molecular weight in the ionic liquid oligomers results in faster dye regeneration and lower charge transfer resistance of I_3_^−^ reduction leading to the improvement of DSSC performance. However, the main limiting factors in the DSSC based on ionic liquids comparing with the conventional wet-type DSSC are the higher recombination and lower injection of charge. At low temperatures, the higher diffusion resistance in the ionic liquid may also be the main limiting factor through its effect to the fill factor [35]. The non-volatile character of ionic liquids also offers the easy packaging for printable DSSCs. Plastic and solid state DSSCs incorporating single walled carbon nanotubes (SWNTs) and imidazorium iodide derivative have been fabricated [36]. The introduction of carbon nanotubes will improve the solar cell performance through reduction of the series resistance. TiO_2_ coated carbon nanotubes (CNTs) were recently used in DSSCs. Compared with a conventional TiO_2_ cell, the TiO_2_-CNT (0.1 wt%) cell gives an increase to short circuit current density (J_SC_), which results in ~50% increase in conversion efficiency from 3.32% to 4.97% [37]. It is supposed that the enhancement of J_SC_ is due to improvement in interconnectivity between the TiO_2_ particles and the TiO_2_-CNTs in the porous TiO_2_ film. When employing SWNTs as conducting scaffolds in a TiO_2_ based DSSC, the photoconversion efficiency can be boosted by a factor of 2 [38]. In absence of SWNT network, a maximum internal photon-current efficiency (IPCE) of 7.36% (350 nm) at 0 V (*vs.* SCE) was observed. The IPCE was enhanced significantly to 16% when the SWNT scaffolds support the TiO_2_ pariticles. TiO_2_ nanoparticles were dispersed on SWNT films to improve photoinduced charge separation and transport of carriers to the collecting electrode surface. Another type of carbon nanomaterial, graphene, was also introduced to the study of DSSC recently. Transparent, conductive, and ultrathin graphene films, as an alternative to the ubiquitously employed metal oxides window electrodes are used for solid-state DSSCs [39].These graphene films are fabricated from exfoliated graphite oxide, followed by thermal reduction. The obtained films exhibit a high conductivity of 550 S/cm and a transparency of more than 70% over 1,000–3,000 nm. Furthermore, they show high chemical and thermal stabilities as well as an ultrasmooth surface with tunable wettability.

A strong increase in energy conversion efficiency could also be observed when tertiary butylpyridine was introduced into the matrix of the organic hole conductor [40] with similar effects for classic DSSC with electrolyte/TiO2 junctions [15]. The increase in V_oc_ may be due to either a charging of surface states or a shift of the conduction band edge [41]. Lithium ion interactions into TiO_2_-B nanowires [42], nanocrystalline rutile TiO_2_ particles [43] and a class of perovskite based lithium ion conductors [44] have been reported. Photovoltages of nanoporous TiO_2_ based DSSC was found to be improved by up 200 mV with a negligible decrease in photocurrent by treating TiO_2_ electrodes with intercalation of Li^+^ [45]. The enhancement of photovoltage is explained in terms of the formation of a dipole layer due to adsorption of Li^+^ on the TiO_2_ surface generated by the reaction of intercalated Li atoms with moisture in air. Addition of lithium salt Li[(CF_3_SO_2_)_2_N] to the spin coating solution of the hole conductor also resulted in a strong performance increase in the final device. The underlying mechanism remained unidentified although charge screening due to partial ionic mobility inside the hole conductor matrix and/or the effect of the present lithium ions on the flat band potential of TiO_2_ were postulated as possible mechanisms [46].

Other n-type semiconducting electrodes besides TiO_2_ have been probed for DSSC. The best studied of the alternative materials to TiO_2_ is ZnO [47–49]. ZnO has similar band gap (3.2 eV) and band edge position to TiO_2_ [50] with similar or smaller crystallite sizes than for typical TiO_2_. The fabrication of DSSC with a branched structure of ZnO nanowires was recently reported [51]. ZnO nanoparticles and nanowires have been used enabling lower temperature manufabricated DSSC electrodes [52,53]. Unlike TiO2, ZnO does not need high-temperature annealing process and extends the electrodes to flexible polymer substrates. The striking optical properties of nanoporous silicon obtained by photoanodic etching [54] extended the materials research scope of photoelectrochemistry to other porous crystalline semiconductors [55]. At present, there is a considerable effort being devoted to DSSC with nanoporous photoanodes [9,56]. Nanoporous semiconductor electrodes were further investigated within the scope of quantum dots. Photoelectrochemical activity has been shown when the quantum dots such as CdS and PbS are attached to a metal electrode in a sub-monolayer array [57–61]. An ordered or disordered monolayer/sub-monolayer of nanometer-sized semiconductor particles (e.g., PbS quantum dots) can be attached to a conducting substrate either by directly or *via* a self-assembled organic monolayer [62,63]. Photoelectrochemical study of organic-inorganic hybrid thin films *via* electrostatic layer by layer assembly was reported [64]. This provides a new way to produce nanoporous semiconductor electrodes for DSSCs.

## 3. Conclusions

Solid state and printable DSSCs will have a promising future for the development of efficient and flexible optoelectronics. Even though DSSCs have lower light to electricity conversion efficiency than the best thin film Si solar cells, they are considerably cheaper to be made and feasible to be printed on flexible substrate. Amorphous Si thin-film cells degrade in sunlight over time, and their efficiencies also go down if the sunlight hits them at some special incident angle. DSSCs are longer lasting and work at wide angles. In addition, DSSCs work more efficiently in indoor light, because the dye absorbs diffuse sunlight as well as fluorescent lighting. With improvements on nonvolatile electrolytes, organic dyes and nanoporous semiconducting electrode, cheaper but more robust DSSCs will definitely take their share in the solar cell markets competing with the traditional thin film solar technologies.
